# Nature’s Arsenal: Uncovering Antibacterial Agents Against Antimicrobial Resistance

**DOI:** 10.3390/antibiotics14030253

**Published:** 2025-03-01

**Authors:** Ina Gajic, Dusan Kekic, Marko Jankovic, Nina Tomic, Mila Skoric, Milos Petrovic, Dragana Mitic Culafic, Natasa Opavski, Petar Ristivojevic, Maja Krstic Ristivojevic, Bojana Lukovic

**Affiliations:** 1 Institute of Microbiology and Immunology, Faculty of Medicine, University of Belgrade, 11000 Belgrade, Serbia; dusan_vk@yahoo.com (D.K.); jankovic.marko1987@gmail.com (M.J.); milaskoric192@gmail.com (M.S.); natasaopavski@gmail.com (N.O.); 2 Group for Biomedical Engineering and Nanobiotechnology, Institute of Technical Sciences of SASA, Kneza Mihaila 35/IV, 11000 Belgrade, Serbia; nina.tomic@itn.sanu.ac.rs; 3 University Clinical Hospital Center “Dr. Dragisa Misovic-Dedinje”, Heroja Milana Tepića, 1, 11040 Belgrade, Serbia; milos.m.petrovic@outlook.com; 4 Faculty of Biology, University of Belgrade, 11000 Belgrade, Serbia; mdragana@bio.bg.ac.rs; 5 Department of Analytical Chemistry, Faculty of Chemistry, University of Belgrade, Studentski trg 12-16, 11158 Belgrade, Serbia; p.ristivojevic@gmail.com; 6 Department of Biochemistry, Faculty of Chemistry, University of Belgrade, Studentski trg 12-16, 11158 Belgrade, Serbia; krstic_maja@chem.bg.ac.rs; 7 College of Health Sciences, Academy of Applied Studies Belgrade, 11000 Belgrade, Serbia

**Keywords:** natural antibacterial agents, antimicrobial resistance, phage therapy, phytochemicals, nanoparticles

## Abstract

**Background/Objectives:** Antimicrobial resistance (AMR) poses a significant public health threat, leading to increased mortality. The World Health Organization has established a priority list highlighting critical multidrug-resistant (MDR) pathogens that demand urgent research on antimicrobial treatments. Considering this and the fact that new antibiotics are only sporadically approved, natural antibacterial agents have seen a resurgence in interest as potential alternatives to conventional antibiotics and chemotherapeutics. Natural antibacterials, derived from microorganisms, higher fungi, plants, animals, natural minerals, and food sources, offer diverse mechanisms of action against MDR pathogens. Here, we present a comprehensive summary of antibacterial agents from natural sources, including a brief history of their application and highlighting key strategies for using microorganisms (microbiopredators, such as bacteriophages), plant extracts and essential oils, minerals (e.g., silver and copper), as well as compounds of animal origin, such as milk or even venoms. The review also addresses the role of prebiotics, probiotics, and antimicrobial peptides, as well as novel formulations such as nanoparticles. The mechanisms of action of these compounds, such as terpenoids, alkaloids, and phenolic compounds, are explored alongside the challenges for their application, e.g., extraction, formulation, and pharmacokinetics. **Conclusions:** Future research should focus on developing eco-friendly, sustainable antimicrobial agents and validating their safety and efficacy through clinical trials. Clear regulatory frameworks are essential for integrating these agents into clinical practice. Despite challenges, natural sources offer transformative potential for combating AMR and promoting sustainable health solutions.

## 1. Introduction

### 1.1. Antimicrobial Resistance Crisis

The rising threat of multidrug-resistant (MDR) bacteria has become one of the top three major public health threats, as stated by the World Health Organization (WHO) [1]. Infections caused by MDR pathogens render standard treatments ineffective, leading to prolonged morbidity, increased mortality, and skyrocketing healthcare costs. Recent projections indicate that by 2050, there could be approximately 1.91 million deaths attributed to antimicrobial resistance (AMR) and 8.22 million deaths associated with AMR worldwide [2]. The alarming increase in antimicrobial resistance highlights the urgent need to assess new antimicrobial agents and their effectiveness against MDR strains.

The most dangerous bacteria, known for their ability to evade antibiotics, are grouped under the acronym ESKAPEE (*Enterococcus faecium*, *Staphylococcus aureus*, *Klebsiella pneumoniae*, *Acinetobacter baumannii*, *Pseudomonas aeruginosa*, *Enterobacter* spp., and *Escherichia coli*). Additionally, the WHO has categorized these pathogens based on their priority for the development of new antibiotics, thereby providing direction for research into innovative antibacterial agents [3].

### 1.2. Natural Antibacterial Agents

**Figure 1:**
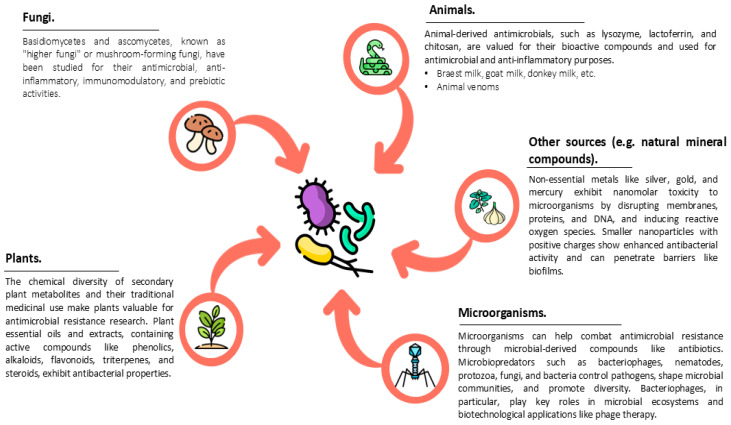
Overview of natural sources of antibacterial agents. Each circle represents a natural source of antibacterial agents (e.g., animals, fungi, microorganisms, plants, and other sources), accompanied by a brief summary of the key facts associated with each source.

Natural antibacterial agents are substances derived directly from nature, both organic (from sources such as plants, microorganisms, or animals) and inorganic (e.g., minerals) in origin, that have the ability to inhibit the growth of bacteria or kill them (Figure 1).

To date, approximately 25,000 natural antibacterial compounds have been identified, contributing to an estimated total of 65,000 to 70,000 natural antibacterial agents. By definition, antibiotics are compounds produced by a living organism, generally a microorganism, that are detrimental to bacteria. As such, they are natural antibacterial agents, although they can be chemically modified to enhance their properties, such as prolonging their elimination time. To date, it has been revealed that microorganisms produce approximately 40,000 antibiotics. In a broader sense, the term “antibiotic” is often used to describe semi-synthetic and synthetic compounds that target bacteria. However, this usage is not accurate, as these compounds are classified as chemotherapeutics and are not derived from natural sources. The number of semi-synthetic and synthetic derivatives developed based on natural compounds has been projected to be around 100,000 [4]. Despite this vast array of compounds, only a few hundred are utilized in clinical practice. The exploration of natural products still holds significant potential for discovering new options to overcome resistance to commonly used and overused antibiotics.

### 1.3. Antibacterial Usage: A Historical Overview

Historical evidence strongly suggests that ancient civilizations relied on a wide range of natural remedies for treating infections. These remedies included herbs, honey, and various animal-derived substances, such as secretions (e.g., venom, milk, cocoons, blood, bile) and excreta (e.g., urine, feces) [5]. Herbal treatments like tea tree oil, different species of basil, garlic, cinnamon, oregano, myrrh, and thyme have been widely used for centuries and remain integral to traditional medicine practices. Today, their active compounds and mechanisms of action are the focus of extensive scientific research. Molds were commonly used as healing agents across various cultures worldwide long before Fleming’s discovery of penicillin in 1928. For instance, Imhotep, an ancient Egyptian healer, employed moldy bread to treat facial infections [6]. Although it is commonly believed that antibiotic exposure is a phenomenon of the modern antibiotic era, traces of tetracycline have been found in human skeletal remains from ancient Sudanese Nubia, dating back 1670 years, as well as in skeletons from the late Roman period in Egypt’s Dakhleh Oasis [7,8]. These findings suggest that dietary exposure to tetracycline-containing materials may have offered protective benefits, as evidenced by low rates of infectious diseases and the absence of bone infections in these populations [9].

## 2. Microorganisms as a Powerful Source of Antibacterial Activity

Table 1 lists frequently used antibiotics, the microorganisms that produce them, the years of their discovery and initial use, and their indications.

In addition to well-known antibiotics, microorganisms can be utilized in various other ways for antibacterial purposes. For example, natural antimicrobials derived from microbial sources include nisin, natamycin, diplococcin, acidophilin, and pediocins. Most of these are cationic, amphiphilic, membrane-permeabilizing peptides (bacteriocins) produced by Gram-positive bacteria.

### 2.1. Modulators of Microbiota

**Figure 2:**
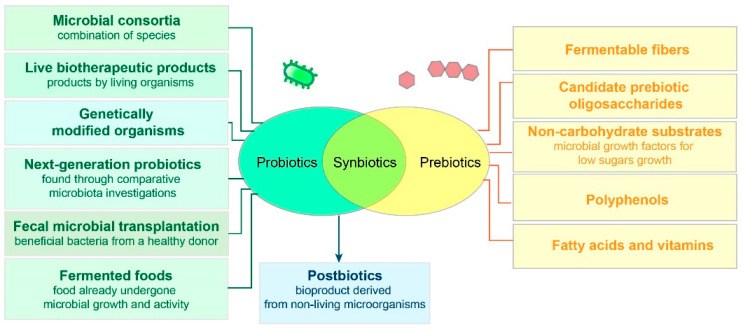
Strategies for modulating gut microbiota to improve health outcomes: probiotics, prebiotics, and synbiotics. Scheme adapted from [12]. The key facts related to probiotics are highlighted in green; the important facts about prebiotics are highlighted in yellow.

Microorganisms present in the gastrointestinal tract take part in many processes important for the host, such as nutrient and drug absorption, vitamin production, regulation of metabolism, immune response, defense against pathogens, and even neural communication [11,12]. Thus, there are potential health benefits from the sensible modulation of microbiota, which can be achieved through various approaches (Figure 2).

Probiotics are live beneficial microorganisms, most notably those belonging to the genera *Bifidobacterium* and *Lactobacillus*. Prebiotics are food components that support the growth of beneficial bacteria. Some, such as inulin or fructooligosaccharides, have already shown potential in clinical studies. The synergistic combinations of prebiotics and probiotics are known as synbiotics. Live therapeutics also include live biotherapeutic products, genetically modified organisms, fecal microbial transplants, and microbial consortia. Postbiotics, on the other hand, are bioactive compounds produced by probiotics (beneficial microorganisms) during metabolic processes. These compounds are typically non-living byproducts or metabolites, such as peptides, lipids, organic acids, polysaccharides, vitamins, antimicrobial peptides (AMPs), short-chain fatty acids, their derivatives, etc., which can have beneficial effects on human health [11,12].

The use of these agents offers advantages due to their multiple mechanisms of action. For instance, probiotic strains help alleviate *Clostridioides difficile*-associated diarrhea (CDAD) by affecting host immune regulation, blocking toxin-binding sites, secreting proteases that directly degrade toxin A, and potentially inhibiting its quorum sensing (QS) system [13,14].

Many microbiota modulators, especially probiotics, are already available as health supplements or adjuncts to antibiotics, or are currently undergoing clinical trials [14,15]. They aim to improve outcomes for over 700 various diseases and conditions, most often gastrointestinal infections and disorders, but also including bacterial vaginosis, mental illnesses, malignancies, and others. However, their efficacy depends on various factors, such as strain selection, dosage, and individual patient characteristics [16]. In certain cases, a lower dose may be equally effective or even more effective than a higher dose, while others may require higher amounts (50 billion+ CFU) to achieve the desired results [17]. Furthermore, it is still unclear whether the strain combinations and large CFU numbers improve efficacy [18]. The absence of a clear dose–response relationship and the lack of information about the exact mechanisms of action imply that more research incorporating modern techniques, such as advanced sequencing and machine learning [11,12], is still needed to allow for more effective use of microbiota modulators as therapeutics [17].

### 2.2. Microbiopredators

Microbiopredators are microorganisms that prey on other microorganisms. The major groups include nematodes, protozoa, fungi, bacteria, and bacteriophages.

Protozoa, such as *Acanthamoeba*, *Tetrahymena*, and *Paramecium,* engulf and digest bacteria and other microorganisms through phagocytosis.

Predatory bacteria prey on various microbes and hunt for and kill other microorganisms, including bacteria, fungi, and even protists [19,20,21]. Predatory bacteria have developed two main types of predation strategies: endobiotic (direct invasion) and epibiotic, based on their interactions with the prey [22]. The endobiotic strategy is employed by small predators that invade the periplasm or cytoplasm of their prey, consuming intracellular macromolecules for nutrition, which supports their growth and division. An example of this strategy is seen in *Bdellovibrio bacteriovorus.* The epibiotic strategy involves predators killing their prey and consuming their nutrients externally. This strategy includes two sub-strategies: solitary predation and group attack. In solitary predation, predators attach to the prey’s surface, killing it and absorbing its nutrients. Examples include *Micavibrio* spp., *Vampirococcus* spp., *Vampirovibrio* spp., *Bdellovibrio exovorus*, and *Myxococcus xanthus* [23]. Both myxobacteria and *Bdellovibrio* spp. prey on a wide range of medically significant organisms, including ”ESKAPEE” pathogens [24,25,26,27,28,29,30,31]. Research into using predatory bacteria as an alternative to antibiotics has primarily focused on *B. bacteriovorus*. As mentioned, these bacteria consume prey cell components and prevent the release of toxic substances, unlike conventional antibiotics or other possible therapeutic approaches, such as using bacteriophages.

In general, microbiopredators can shape microbial communities by controlling bacterial populations, preventing the overgrowth of harmful pathogens, and promoting microbial diversity. Additionally, in agriculture, they can play an essential role in promoting soil health by regulating microbial community structure and limiting harmful plant pathogens. However, the potential for unforeseen ecological consequences needs to be addressed. Notably, bacteriophages are the microbes that have seen the most progress in both research and application.

### 2.3. Phages—Tiny Solution for a Big Problem?

Bacteriophages, or phages, are ubiquitous viruses that can infect only bacteria. In 1915, Frederick Twort first discovered the effects of bacteriophages; two years later, Félix d’Hérelle conducted further investigations, eventually naming these microorganisms [32]. Bacteriophages, once considered a nuisance due to their role in causing fermentation failures in cheese-making and other industrial processes, are now increasingly recognized as promising antimicrobials capable of combating pathogenic bacteria. Remarkably, phages reduce bacterial biomass by about 40% each day [33]. The current renewed interest in phage biology has been fueled by whole-genome sequencing projects that further reveal the presence of phage DNA residing in the genomes of their bacterial prey. Moreover, the impetus behind the “weaponization” of bacteriophages is the dramatic rise of antibiotic resistance in pathogenic bacteria, the alarming rate of infections caused by MDR bacteria, and biofilm-associated infections. In addition to direct curative interventions, the use of phage biocontrol to disinfect equipment and surfaces in the food industry presents itself as an exciting prospect [34].

Although still not widely used in direct therapy, there is a steady advance in research addressing the utilization of phages in clinical settings. A recent comprehensive review on phage therapy highlights key clinical trials and literature reviews that began gaining momentum only in the late 1990s [35,36]. A critical review of bacteriophage therapy in both adults and neonates revealed an efficacy of 87% and a safety rate of 67% for the phages tested, targeting a range of infections caused by ESKAPE bacteria, including skin, eye/ear, blood, gastrointestinal/urinary, and solid organ infections. The authors evaluated either individual phages or phage cocktails, with applications delivered via topical, oral, rectal, intravenous, intracavitary, direct organ instillation, and inhalation methods. However, only a limited number of studies examined phage resistance (35%) [37]. The bacteria’s susceptibility to bacteriophages varied depending on the bacterial species or type, the type of phage or cocktail used, and the patient’s individual response. While the majority of studies report clinical success, a few have noted failures, emphasizing the need for further clinical research to validate the effectiveness of phage therapy—potentially not as a universal solution but as a means of targeting specific bacterial strains [37,38].

Bacterial virus research primarily focuses on preventing and treating infections caused by multidrug-resistant bacteria, such as *Acinetobacter* spp. [38]. There are certainly several advantages to using phages in infection prevention and treatment. They are ubiquitous, have a relatively innocuous profile for human use, and can be rapidly adapted to fight emergent infections [39]. Bacteriophages exhibit strong selectivity, specifically targeting and eliminating certain types of bacteria. It is worth noting that, while this precision is advantageous, it also poses a challenge, as choosing the right phage for the intended bacteria is crucial. Finally, phages are on the horizon even in personalized therapy endeavors for intractable infections [40].

Apart from many apparent benefits, there are certain caveats to the use of bacteriophages, not the least of which are universally accepted usage regulations. The effectiveness of phage-based products for a given disease may vary, influenced by climatic factors like temperature [41]. Moreover, bacteria may evolve resistance to phages by utilizing various protection mechanisms [42]. The prevention of phage attachment or release, receptor removal or modification, and blocking bacteriophage DNA injection are all examples of situations in which phage intervention might fail [41]. Here also lies a continuous hurdle for developing generalized phage therapy—the prospect of viruses exerting selection on specifically targeted bacteria, which elicits progression into phage resistance at some point during the therapy [40]. In this context, medicine aims to employ two key harmonizing strategies to reduce bacterial resistance: 1) to minimize the potential for microbe populations to develop phage resistance and 2) to guide the evolution of phage-resistant bacteria in the direction of clinically positive results. The dark side of phages may result from their impact on beneficial bacteria that are part of the microbiota or from contaminating areas that contain useful microorganisms; for example, phage contamination may result in the destruction of bacteria used in industry. Finally, the cost of employing phage therapy may prove to be high [43].

So far, the Food and Drug Administration (FDA) has not approved phage therapies, although clinical trials are underway, and compassionate use can at times be approved. In the United States of America (USA), phages are categorized as pharmaceuticals, while in the European Union (EU), they are categorized as medicinal products [44]. They must have marketing and manufacturing authorization from the European Medicines Agency (EMA) in the EU and the FDA in the USA, just like conventional medications. Phage treatments must undergo preclinical in vitro and in vivo validation before they can be approved for use in humans. Phase I to IV clinical trials must then be conducted after preclinical tests are finished in order to assess their safety and effectiveness in people. Phage therapies have not yet advanced to phase IV of clinical trials [35], although they offer a promising solution to the growing challenge of antibiotic resistance. With their precision in targeting harmful bacteria, they hold the potential to revolutionize treatments, offering hope for more effective and sustainable options in the fight against infections.

Although the bacteriophage approach to combating harmful pathogens is a groundbreaking idea, significant effort is still required to harness these viruses for medical and biotechnological applications.

## 3. Higher Fungi-Derived Antibacterial Agents

Among fungi, basidiomycetes and ascomycetes, referred to as “higher fungi” (i.e., mushroom-forming fungi), have historically been explored for various beneficial properties, including medicinal ones [45], such as antimicrobial, anti-inflammatory, immunomodulatory, antidiabetic, cytotoxic, antioxidant, hepatoprotective, anticancer, antioxidant, antiallergic, antihyperlipidemic, and prebiotic activities [46]. Mycochemicals are bioactive metabolites present in the mycelium and fruiting bodies of mushrooms. Structurally, these substances can be categorized into the following groups: polysaccharides, terpenoids (derived by adding functional groups to terpenes), phenolic compounds, polyunsaturated fatty acids, lipids, glycoproteins, polyketides, steroids, alkaloids, anthraquinones, quinolones, benzoic acid derivatives, oxalic acid, peptides, proteins, and unknown, unidentified metabolites [47].

Their antibacterial activity has been shown to be quite diverse, affecting both Gram-positive and Gram-negative bacteria. Certain mushroom-derived compounds showed activity against *Streptococcus pneumoniae* [48], *Bacillus subtilis* [49], *E. coli*, *K. pneumoniae*, and *P. aeruginosa* [50]. Numerous examples of mushroom-derived compounds that can interfere with the formation of biofilms exist. Thus, the in vitro antibiofilm activity of *A. auricula-judae* extracts against *P. aeruginosa*, *E. coli*, and *S. aureus* has been demonstrated [51]. Specifically, coprinuslactone, isolated from the fruiting body of the edible mushroom *Coprinus comatus* [52], dispersed biofilms of *P. aeruginosa* and damaged *S. aureus* cells in biofilms by inhibiting UDP-N-acetylglucosamine enolpyruvyl transferase (MurA), which is essential for bacterial cell wall synthesis, while certain anthocyanidins (pelargonidin, cyanidin and delphinidin), a class of flavonoid compounds, affect *P. aeruginosa* [53]. The aqueous extract from *B. edulis* achieved a reduction of over 94% in *E. coli* biofilm [54,55], whereas ethanolic extracts of *Russula* spp. exhibited antibiofilm activity against *S. aureus*, inhibiting biofilm formation by more than 80% [56]. The mechanisms by which the antibacterial effects are achieved are relatively unclear but may be related to the inhibition of cell wall synthesis, interference with specific microbial metabolic processes, alteration of signal transduction, modification of gene expression pathways, disruption of the redox balance, with consequent oxidative stress and cell death, disruption of the integrity of the bacterial cell membrane, and interference with QS [57]. To the best of our knowledge, the mechanisms of action of mushroom-derived phytochemicals as antibacterial agents have not been systematically explored, and existing data are mostly phenomenological. This lack of information leads to a shortage of clinical trials assessing the benefits of mushroom-derived antimicrobials for treating human patients.

Altogether, mushrooms present an insufficiently explored reservoir of substances with promising antibacterial properties. Ongoing research will hopefully lead to the identification of candidates suitable for further exploration by the pharmaceutical industry. However, despite their potential, fungal-derived substances are currently far from achieving clinical application as approved antimicrobial agents.

## 4. Plant- and Endophyte-Derived Antibacterial Agents

Due to the extensive chemical diversity of secondary plant metabolites, coupled with their longstanding use in traditional medicine, plants can serve as highly valuable natural reservoirs for research focused on AMR [58]. Numerous authors across the world have documented the application of plants and herbs, including their derivatives, as an innovative therapeutic strategy in a currently very challenging field—the treatment of infectious diseases [59]. Multiple studies report that phytochemicals have the capacity to exhibit antibacterial activity against both sensitive and resistant pathogens through diverse mechanisms of action, including biofilm disruption and QS inhibition [60]. The section below highlights various plant products and the plant species from which they are derived, along with their potential therapeutic applications in the treatment of numerous microbial infections.

### 4.1. Antimicrobial Properties of Essential Oils and Plant Extracts

Essential oils (EOs) and plant extracts are rich sources of bioactive compounds with significant antimicrobial potential. EOs are secondary metabolites produced by aromatic plants, comprising a complex mixture of terpenes, aldehydes, alcohols, ethers, and phenols, as listed in Table 2 [61,62]. These volatile and aromatic molecules exhibit low solubility in water and can be extracted using various techniques. Due to their complex composition, the development of resistance to these molecules is significantly limited [62]. Several plant species, including mint, clove, sage, thyme, lavender, cinnamon, coriander, oregano, and rosemary, have demonstrated strong antibacterial and antifungal activities, potentially through the disruption of cell wall integrity, interference with metabolic pathways, and alteration of cell membrane potential [63]. Citronellol and carvacrol EOs demonstrated a potent inhibitory effect on *E. coli* growth, potentially by disrupting the integrity of the cell wall via interaction with its components, including membrane proteins [63]. Moreover, it was determined that oregano EO can inhibit bacterial growth and reduce lactic acid production in *Salmonella* Typhimurium, *Yersinia enterocolitica*, and *E. coli* [63]. As reported by Radu et al., essential oils from clove, sesame, cinnamon, lavender, lemongrass, and eucalyptus have shown effectiveness in the treatment of dental cavities and periodontitis, suggesting potential antimicrobial applications of EOs in oral health [64]. As natural products, EOs require rigorous quality evaluation to meet the criteria for the GRAS classification [65,66,67]. Plant extracts also exhibit considerable chemical complexity, often containing hundreds of distinct constituents [68]. Certain active compounds, such as phenolics, alkaloids, flavonoids, triterpenes, and steroids, display intrinsic antibacterial properties and antibiotic resistance-modifying activities [69,70]. While some of these compounds lack direct antibiotic activity, they can enhance the effects of antibiotics when used in combination, contributing to the overcoming of bacterial resistance [69].

Medicinal plants, such as *Salvia officinalis*, *Sambucus nigra*, and *Malva sylvestris*, are recognized for their anti-inflammatory and antimicrobial effects [71]. Studies highlight the potential use of substances from members of the *Asteraceae* family, particularly *Matricaria chamomilla*, for treating infections, while ethanol-based extracts from *Helichrysum* spp. have demonstrated the inhibition of *E. coli* and *S. aureus* [70,71]. According to Chassagne et al., plant families such as *Zingiberaceae*, *Rutaceae*, *Myrtaceae*, *Lauraceae*, and *Rubiaceae* exhibit strong antibacterial activity, showing the lowest mean minimal inhibitory concentration (MIC) among the 50 prominent plant families tested [72]. In a 6-month study, 140 clinical isolates of *P. aeruginosa* were collected from patients in the burn wards. All MDR *P. aeruginosa* strains were inhibited by *Aloe vera* at comparable MIC_50_ and MIC_90_ values of 200 µg/mL [73].

Traditional medicine in Serbia and the Balkans frequently utilizes *Herniaria hirsuta* (hairy rupturewort), *Prunus avium* (wild cherry), *Rubia tinctorum* (common madder), and *Sempervivum tectorum* (common houseleek) for the treatment of bacterial infections [74]. Antimicrobial susceptibility testing (AST) on *E. coli* ATCC and MDR strains confirmed their antibacterial effect. However, it was shown that extraction methods influence efficacy: for *H. hirsuta* and *P. avium*, the ethanol extracts were more effective, while the aqueous extracts of *R. tinctorum* and *S. tectorum* exhibited higher antibacterial potential [75].

Myrrh, traditionally used for its antiseptic and anti-inflammatory properties, demonstrates antimicrobial activity against bacteria, fungi, and viruses. Its phenolic compounds and terpenes disrupt microbial membranes [76,77,78] and demonstrate effectiveness against oral pathogens, such as *Streptococcus pyogenes*, supporting its application in treating tonsillopharyngitis and gingivitis [79].

Ginger, which is rich in bioactive compounds like gingerol, possesses anti-inflammatory, antioxidant, and antimicrobial properties [80]. It has demonstrated efficacy against respiratory and gastrointestinal infections, with ethanolic extracts showing activity against *Salmonella* Typhi and *E. coli*. Gingerol derivatives also inhibit periodontal bacteria, reinforcing their potential as natural antimicrobial agents [81,82,83].

Echinacea is widely recognized for its immunomodulatory effects, which reduce the severity of respiratory infections. Its alkamides and caffeic acid derivatives exhibit antimicrobial properties, supporting its role in immune health and infection management [84].

Elderberry, rich in flavonoids and anthocyanins, has demonstrated strong antiviral and antioxidant activities, showing effectiveness against influenza viruses and bacterial pathogens, including *S. pyogenes* [85]. Additionally, elderflower extracts show antibacterial activity against methicillin-resistant *Staphylococcus aureus* (MRSA) and *Salmonella* spp., making elderberry a valuable natural remedy [86].

These findings highlight the potential of essential oils and plant extracts as natural antimicrobial agents, offering promising alternatives for infection management and resistance mitigation.

### 4.2. Endophytes

Endophytes, which are microorganisms that live within a plant for at least part of their life cycle without causing harm to the host plant, are vital components of the phytomicrobiome. In addition to fungi, archaea, lichens, and algae, approximately 75% of identified bacterial endophytes belong to the genus *Streptomyces*, a group of filamentous, Gram-positive bacteria classified within the *Actinomycetia* class. These microorganisms exhibit significant potential as biocontrol agents due to their diverse functional properties [87]. Their use is primarily driven by the need to reduce reliance on synthetic pesticides, mitigate the emergence of herbicide- and pesticide-resistant pathogenic microorganisms, and address the increasing occurrence of natural disasters associated with global climate change [88]. However, extensive research highlights the potential of endophytic microorganisms and their bioactive metabolites in the development of strategies to treat human pathogens. Hence, coumarins, such as 5,7-dimethoxy-4-p-methoxylphenylcoumarin and 5,7-dimethoxy-4-phenylcoumarin, produced by the endophytes *Streptomyces aureofaciens* and *Ampelomyces* spp. isolated from *Urospermum picroides* (prickly goldenfleece) and *Zingiber officinale* (ginger), have been shown to exhibit antibacterial activity against *S. aureus*, *S. epidermidis*, and *E. faecalis* [89]. The emerging data underscore the multifaceted applications of endophytes, highlighting their potential not only in agriculture but also in the medical and biotechnological fields [87].

### 4.3. Phytochemicals with Antimicrobial Activities, Extraction Methods, and Antimicrobial Susceptibility Testing

Phytochemicals can be divided into several major classes based on their chemical structures, botanical origins, biosynthesis pathways, or biological properties. The most widely accepted phytochemical classification scheme is based on their chemical structures (e.g., phenolics, alkaloids, saponins, terpenoids, limonoids, polyacetylenes, secoiridoids, etc.) [90]. Numerous studies have been conducted in vitro and in vivo in recent years to assess the efficacy of plant phytochemicals as antibacterial agents (Table 2). As phenolic compounds, flavonoids are the most abundant secondary metabolites in different plant species and are effective antibacterial agents. They disrupt the processes of biofilm formation, cell envelope and nucleic acid synthesis, and inhibit the electron transport chain, ATP synthesis, bacterial metal enzymes, and bacterial toxin function. Flavonoids also act as antibiotic resistance reversal agents or antibiotic potentiators through the inhibition of bacterial efflux pumps [91].

The extraction of antimicrobials from plants is essential for developing natural preservatives, pharmaceuticals, and functional foods. Traditional methods, such as liquid–liquid extraction (LLE), maceration, percolation, reflux, and Soxhlet extraction, are widely used but often require large volumes of organic solvents and prolonged extraction times, which may degrade heat-sensitive antimicrobials. To overcome these challenges, extensive efforts have been dedicated to developing more efficient and sustainable green extraction techniques, such as ultrasound-assisted extraction (UAE), microwave-assisted extraction (MAE), supercritical fluid extraction (SFE), and pulsed electric field (PEF) extraction. UAE and MAE improve efficiency by disrupting plant cell walls, leading to enhanced recovery of antimicrobial compounds with reduced solvent usage and shorter processing times. SFE, which uses supercritical CO₂, provides selective extraction without toxic residues but requires specialized equipment and incurs high operational costs. While modern techniques enhance yield and selectivity, challenges such as scalability, cost, and antimicrobial stability must be addressed to ensure industrial application [92,93]. Extraction is followed by classification and quantification using spectrophotometry, gas chromatography, high-performance liquid chromatography, or capillary electrophoresis methods [94].

A limited number of AST methods have been effectively applied to evaluate the antibacterial activity of natural products like phenolic compounds. The disc diffusion assay is widely utilized, particularly for natural substances with low molecular weight. However, the reliability and reproducibility of this technique depend on several critical parameters, and one of the major disadvantages is that it is qualitative and does not distinguish between bactericidal and bacteriostatic effects. Considering the tendency of natural products to adsorb onto the hydrophilic surface of the disc, which limits their diffusion into the medium, a more sensitive method for AST of plant-derived high molecular weight compounds is the agar well diffusion assay [95]. Both of the aforementioned qualitative methods are increasingly being replaced by more precise and reproducible quantitative dilution methods—specifically, the agar or broth dilution method—with particular emphasis on the broth microdilution method as the most reliable approach for determining the true potency of a pure compound [71,96,97].

Using conventional or molecular techniques, it is possible to define the antibacterial activity of different phytochemicals, like minimal inhibitory or bactericidal concentrations, synergistic activity, effects on efflux pumps, bacterial enzymes, virulence factors, or biofilm production, and the plasmid curing process (Table 2).

However, only a few plant-derived antibacterial drugs are in clinical trials or on the market, mostly targeting urinary tract infections (UTIs) and *H. pylori* infections [98]. Nevertheless, there are numerous studies conducted on cell lines or in animal models [99]. Although the primary advantages of medicinal plants and higher fungi include their demonstrated efficacy, minimal incidence of adverse effects, cost-effectiveness, and widespread accessibility, antimicrobial testing of plant- and fungi-derived compounds is challenged by the absence of standardized methods for defining and comparing the susceptibility of bacteria to these compounds [95,100]. Additionally, it is important to account for the possibility that the composition of particular essential oils and extracts from different regions may vary, influenced by environmental factors (ecology, climate, geography), the plant developmental stage, soil properties (texture, acidity), and the plant’s genotype or subspecies [101,102].

**Table 2:** Overview of phytocompounds with antibacterial activity categorized by their chemical class, plant source, target bacteria, and mechanism of action.

Chemical Class	Phytochemical	Source	Target Bacteria	Mechanism of Action	Reference
Flavonoids	Catechin	Green tea (*Camellia sinensis*)	MDR *P. aeruginosa*	Aztreonam reversal agent	[103]
		*Canarium patentinervium*	*E. coli*	AcrAB-TolC efflux pump (*acrA* gene) inhibition with biofilm reduction; synergy with tetracycline; bactericidal activity	[104]
	Theaflavin-3,3′-digallate	*Camellia sinensis*	MRSA	MBL inhibitor (binding to Gln242 and Ser369); synergy with penicillins and cephalosporins	[105]
	Quercetin	Tomatoes, grapes, onions, etc.	CRAB, CRPA	Synergy with meropenem; bactericidal activity; *bla_NDM_* and *AdeB* gene inhibition; disruption of cell wall/membrane integrity	[106]
	Kaempferol	Delphinium, witch hazel, grapefruit, etc.	Colistin-resistant Gram- bacteria (*P. aeruginosa*, *E. coli*, *A. baumannii*, *K. pneumoniae)*	Synergy with colistin; antibiofilm effects; bactericidal activity; disruption of cell membrane integrity	[107]
	Pinostrobin	Finger Root	*P. aeruginosa* *E. coli*	Efflux pump inhibition (ciprofloxacin potentiator)	[108]
	Licochalcone A	*Glycyrrhiza* species	*E. faecalis*	Bactericidal activity on planktonic cells through intracellular signal transduction/transcriptional regulation; reduced production of persister cells; antibiofilm effects through *agg*, *esp*, and *srtA* gene inhibition	[109]
	Proanthocyanidin	Cranberry	*P. aeruginosa*	Decrease in swarming motility and biofilm production; Down-regulation of cytochrome C and acetyl-CoA enzyme; Gentamicin potentiator	[110]
Alkaloids	Berberine	*Berberis* species, *Hydrastis canadensis*	MDR Gram - bacteria, including *E. coli* MRSA, *C. difficile* *M. abscessus*, *M. avium*	Synergy with various antibiotics due to inhibition of antibiotic efflux; disruption of biofilm formation; regulation of host immunity and gut microbiota	[111]
	Reserpine	*Rauwolfia* *serpentina*	*S. maltophilia*	Efflux pump inhibition (fluoroquinolones potentiator)	[112]
	Piperine	*Piper nigrum* *Piper longum*	CRPA	MexAB-OprM efflux pump inhibition (up-regulation of *MexR* gene/down-regulation of *MexA*, *MexB*, and *OprM* gene expressions); synergy with imipenem	[69]
	Tomatidine	Solanaceous plants	*S. aureus*	ATP synthase inhibitor	[113]
Terpens	8-epidiosbulbin E-acetate	*Dioscorea bulbifera*	VRE VRSA *P. aeruginosa* *E. coli* *S. sonnei* *S.* Typhi	Plasmid curing	[114]
	Thymol	*Thymus capitatus*	Colistin-resistant *P. aeruginosa*, *E. coli*, *E. cloacae*, *K. pneumoniae*	Cell membrane damage; Antibiofilm effects; Synergy with colistin	[69]
	Curcumin	*Curcuma longa*	*P. aeruginosa* *E. coli* *P. mirabilis* *S. marcescens*	Quorum sensing Inhibition and biofilm formation	[115]
	Obacunone	Grapefruit seed	EHEC	Quorum sensing Inhibition and biofilm formation; TTSS inhibition	[116]
	Eugenol	Essential oils (clove oil)	MRSA	Cell membrane damage; Decreasing the expression of biofilm-and enterotoxin-related genes	[117]
	Farnesol	Essential oils	Colistin-resistant Gram - bacteria	Cell membrane damage; Antibiofilm effects; Synergy with colistin	[63]
Organosulfurs	Allicin	*Allium sativum *	G- and G+ bacteria, including *E. coli, P. aeruginosa*, MRSA	DNA gyrase inhibition; cell membrane disruption	[118,119,120]
	Ajoene	*Allium sativum *	*P. aeruginosa*	Quorum sensing inhibition	[121]
Stilbenes	Resveratrol-derived stilbenoids	Grapes Peanuts Cranberries	*L. monocytogenes* *S. aureus* *E. faecium* *E. faecalis* *B. cereus*	Cell membrane damage	[122]
Coumarins	Simple coumarin Imperatorin Isoimperatorin	*Angelica dahurica *	*P. aeruginosa*	Synergy with ampicillin and ceftazidime; Antibiofilm effects	[123]
	Aegelinol Agasyllin	*Ferulago* *campestris*	*S. aureus* *E. cloacae* *E. aerogenes* *H. pylori*	DNA gyrase inhibitor	[124]
	Galbanic acid	*Ferula szowitsiana*	MRSA	Efflux pump inhibition (ciprofloxacin and tetracycline potentiator)	[125]

MRSA: methicillin-resistant *Staphylococcus aureus*; MBL: metallo-β-lactamases; CRAB: carbapenem-resistant *Acinetobacter baumannii*; *K. pneumoniae*: *Klebsiella pneumoniae*; *P. aeruginosa*: *Pseudomonas aeruginosa*; CRPA: carbapenem-resistant *Pseudomonas aeruginosa*; *E. coli*: *Escherichia coli*; *S. aureus*: *Staphylococcus aureus*; MDR: multidrug-resistant; *C. difficile*: *Clostridioides difficile*; *M. abscessus*: *Mycobacterium abscessus*; *M. avium*: *Mycobacterium avium*; *S. maltophilia*: *Stenotrophomonas maltophilia*; VRE: vancomycin-resistant enterococcus faecalis; VRSA: vancomycin-resistant *Staphylococcus aureus*; *S. sonnei*: *Shigella sonnei*; *S.* Typhi: *Salmonella* Typhi; *P. mirabilis*: *Proteus mirabilis*; *S. marcescens*: *Serratia marcescens*; *E. cloacae*: *Enterobacter cloacae*; *E. aerogenes*: *Enterobacter aerogenes*; *L. monocytogenes*: *Listeria monocytogenes*; *E. faecium*/*faecalis*: *Enterococcus faecium*/*faecalis*; *B. cereus*: *Bacillus cereus*; *H. pylori*: *Helicobacter pylori*; EHEC: Enterohemorrhagic *Escherichia coli*; TTSS: Type three secretion system; G+/G− bacteria: Gram-positive/Gram-negative bacteria

## 5. Animal-Derived Antimicrobial Agents

Examples of natural antimicrobials from animal sources, including foods of animal origin like milk (human breast milk, goat’s milk, donkey’s milk) and eggs, as well as arthropods, crustaceans, and snakes, consist of lysozyme, lactoferrin, lactoperoxidase, chitosan, magainin, pleurocidin, curvacin A, spheniscin, and free fatty acids [126].

### 5.1. Milk and Eggs

Alpha-lactalbumin, lactoferrin, and osteopontin are among the promising components that could lead to the development of antimicrobial agents derived from human breast milk [127]. Additionally, compounds from donkey and goat milk with antibacterial properties against *E. coli* and other bacteria have been confirmed [128,129]. Egg white contains different proportions of antibacterial substances, mainly ovotransferrin, lysozyme, ovomucoid, and ovoid inhibitors [130].

However, the greatest interest in potential antibacterial effects lies in the toxins and enzymes produced by certain animals.

### 5.2. Antibacterial Agents from Venomous Animals

Animal venoms have long been recognized for their bioactive compounds, which are used in traditional medicine for various purposes, including antimicrobial, anti-inflammatory, and anticancer activities. From ancient Greek mythology, the Rod of Asclepius, with a venomous snake coiled around it, symbolizes medicine and healing (pharmacy). Indeed, several drugs have been derived from these venoms, like captopril, ziconotide, eptifibatide, exenatide, etc. [131]. Venomous animals are found in many phyla, including *Chordata* (reptiles, fishes, and amphibians), *Arthropods* (arachnids and insects), *Mollusca* (cone snails), *Echinodermata* (starfishes and sea urchins), and *Cnidarians* (sea anemones, jellyfish, and corals) [132]. Most animal venoms are complex mixtures of biologically active compounds, such as proteins, peptides, enzymes, nucleotides, lipids, biogenic amines, and other unknown substances, which exhibit both cytotoxic and antimicrobial properties. Animal venoms consist of both enzymatic and nonenzymatic components (e.g., proteins/enzymes—phospholipase A2 (PLA2), L-amino acid oxidase (L-AAO), metalloproteinases, AMPs, etc.) [133]. AMPs are produced by all invertebrates, in which they represent a primary defense mechanism, as invertebrates lack an adaptive immune system [134]. Another important characteristic is that due to their diverse mechanisms of action, resistance to AMPs develops much less frequently than to conventional antibiotics [135]. To support research in this area, an Antimicrobial Peptide Database was created [136], which currently consists of 4257 recorded AMPs [137]. It provides detailed information on the sequence, activity, toxicity, and references for each specific AMP.

The studies conducted thus far have focused on testing whole venoms or their fractions to identify active toxin components, or on modifying derived AMPs to enhance their activity or broaden their antimicrobial spectrum. Notably, cytotoxic properties may contribute to the venom’s ability to kill or inhibit the growth of bacteria. Despite their potential therapeutic applications, the cytotoxicity of venoms must be carefully controlled to avoid harm to human cells during therapeutic use. Research is ongoing to isolate and refine these bioactive compounds to harness their antibacterial potential while minimizing their harmful effects on host tissues.

Snake venoms are the most complex and studied animal toxins. As previously mentioned, venoms are mostly composed of enzymes and nonenzymatic proteins/peptides, some of which have antimicrobial effects. They can exhibit antibacterial activity against both Gram-positive and Gram-negative bacteria, either as whole venoms or as fractions. For instance, *Vipera ammodytes ammodytes* venom demonstrated antimicrobial effects against *S. aureus*, *E. faecalis*, *S. pneumoniae*, *E. coli*, *K. pneumoniae*, and *P. aeruginosa* [133,138]. In snakes, the enzymes PLA2 and L-AAO are primarily responsible for antimicrobial activity. PLA2 exerts its bactericidal effect through membrane permeabilization and subsequent activation of the damage pathway, while L-AAO seems to produce hydrogen peroxide and induce oxidative stress in the target cell [139,140]. As an example, the CaTx-II PLA2 enzyme derived from *Crotalus adamanteus* venom manifested MICs for *S. aureus* and *B. pseudomallei* at 7.8 µg/mL and for *E. aerogenes* at 15.6 µg/mL in a mice wound-healing model [141]. The L-AAO derived from *Bothrops marajoensis* venom showed an inhibitory effect on the growth of *P. aeruginosa* and *S. aureus* [142]. As another example, cathelicidins are small AMPs with broad-spectrum, highly effective antibacterial action against both Gram-positive and Gram-negative bacteria. They are more effective than routinely used and tested antibiotics and cause minimal damage to host cells [143]. The most important ones are isolated from *Naja atra* (NA-CATH), *Ophiophagus hannah* (OH-CATH), *Bungarus fasciatus* (BF-CATH), *Hydrophis cyanocinctus* (Hc-CATH), *Sinonatrix annularis* (SA-CATH), *Python bivittatus* (CATHPb1), etc. [144]. These molecules have a very potent activity through their low MIC values, like in *Naja atra* (3.6 µg/mL against *B. thailandensis*) or *Hydrophis cyanocinctus* (2.34 µg/mL and 4.69 µg/mL for *E. coli* and *S. aureus*, respectively) [145,146]. Furthermore, there are waprins and vespryns, two new protein families from snake venoms that exhibit antibacterial activity. Among them is omwaprin, a cationic peptide from *Oxyuranus microlepidotus*, which has selective and dose-dependent activity against Gram-positive bacteria [144]. Also, there is Pep5Bj, a peptide isolated from *Bothrops jararaca*, along with many others that have activities not only against bacteria but also against fungi, viruses, and parasites [131].

Various spider species produce both venom and silk, with some species’ silk demonstrating antibacterial properties (*Pardosa brevivulva* and *Tegenaria domestica*) [147,148]. Spider venoms derived from the ant spider, *Lachesana tarabaevi*, contain a range of antimicrobial peptides (latarcins 1, 2a, 3a, 4b, 5, and cytoinsectotoxin 1a) that significantly reduce the viability of *Chlamydia trachomatis* in infected cells [149]. Lycotoxins are pore-forming peptides, with Lycotoxins-I and -II from *Lycosa carolinensis* being the first AMPs detected to have dual antibacterial and antifungal activities [150]. Acylpolyamine VdTX-I, known for its effective antibacterial properties against *E. coli*, *S. epidermidis*, and *S. aureus,* was isolated from the venom of the tarantula *Vitalius dubius* [151]. Another group of AMPs with membrane lysis activity is cupiennins. Among them, Cupiennin Ia, isolated from the venom of *Cupiennius salei,* exhibits high antibacterial activity against both Gram-positive and Gram-negative ATCC species (*S. aureus*, *E. faecalis*, *E. coli*, *and P. aeruginosa*) with low MIC values [152]. Also, there are Latarcins, derived from the venom of the spider *Lachesana tarabaevi;* Oh-defensin, isolated from the venom of the spider species *Ornithoctonus hainana;* and Gomesin, obtained from the spider *Acanthoscurria gomesiana,* which exhibits highly potent activity against all tested Gram-positive and Gram-negative bacteria [153,154,155]. Slightly modified venom component LyeTxI from the spider *Lycosa erythrognatha* becomes LyeTxI-b, which exhibits improved in vitro and in vivo activity [156].

In traditional Chinese medicine, scorpions have also been used as therapeutics. The initial defensins identified from the hemolymph of *Leiurus quinquestriatus* were effective solely against Gram-positive bacteria. Scorpine was the first broad-spectrum AMP extracted from *Pandinus imperator*, demonstrating low MIC values ranging from 1 to 10 µM [157,158]. Heteroscorpine-1, a peptide obtained from *Heterometrus laoticus*, exhibits extensive antibacterial properties, with effectiveness 300 times greater than that of the entire crude venom [159]. Androctonin is another AMP form derived from *Androctonus australis* venom, exhibiting a significant antibacterial effect [160]. A number of AMPs that lack cysteine residues have also been isolated and purified from scorpion venoms, like hadrurin (*Hadrurus aztectus*), parabutoporin (*Parabuidethus schlechteri*), two isoforms of opistoporin 1 and 2 (*Opistophtalmus carinatus*), vejovine (*Vaejovis mexicanus*), etc. [144]. Another example of upgrading a natural peptide is mucroporin-M1, which is a modification of the original mucroporin derived from *Lychas mucronatus.* This modification resulted in activity against both Gram-positive and Gram-negative bacteria, particularly against antibiotic-resistant forms like MRSA [161].

Although venomous cone snails are not numerous, they produce AMPs with biochemical properties similar to those found in snake, scorpion, and spider venoms. On the other hand, these so-called conopeptides or conotoxins exhibit low and highly specific antimicrobial activity, with only one peptide (Lo6/7a) being active against *Bacillus megaterium* [135,162]. Furthermore, snail slime from *Achatina fulica* contains a potent AMP, mytimacin-AF, which exhibits strong activity against both Gram-positive and Gram-negative bacteria [163]. Also, mucins from *Achatina fulica*, *Archachatina marginata*, and *Helix aspersa* show bactericidal effects against *S. aureus.* The peptides from *Lymnaea stagnalis* exhibit the same effect, with greater potency than ampicillin and chloramphenicol [164,165,166].

Wasps and bees are closely related insect species within the *Hymenoptera* order, known for their complex venoms, which contain peptides, proteins, enzymes, and other small molecules. Some FDA-approved drugs derived from their venoms are already in use [167]. As for the antibacterial effect, their venoms, such as those of *Vespa orientalis* and *Vespa magnifica*, are known to be highly active and exhibit broad-spectrum activity [168,169]. For example, in *Vespa tropica* venom, nine AMPs were identified and classified as mastoparans and vespid chemotactic peptides [170]. Likewise, bee venom from *Apis mellifera* is known for its antibacterial effect, as the purified peptide melittin has proven to be highly active against various bacterial species, including drug-resistant ones [171]. It is worth noting that its activity can be enhanced further via specific chemical modification [172]. Furthermore, other bees’ active peptides, such as secapin and apidermin 2, have also demonstrated antibacterial properties [173,174].

In ants, venom glands and Dufour glands produce compounds with antimicrobial properties, as seen in *Crematogaster scutellaris* [175]. Examples of venom-derived AMPs with high activity and a broad spectrum include pilosulin-1 from *Myrmecia pilosula* and bicarinalin detected in *Tetramorium bicarinatum* venom [176,177].

Additionally, a group of peptides known as ponericins, isolated from *Neoponera goeldii* venom, is classified into three families: Ponericins G, W, and L. These peptides exhibit activity against both Gram-positive and Gram-negative bacteria by targeting their cell membranes. Notably, ponericins W are highly similar to melitin [178].

Frogs and toads have mucous glands spread across their skin that secrete slimy substances, which play various roles in their physiology. Thus, two bufadienolides (marinobufagin and telocinobufagin) displaying antibacterial effects, with MIC values comparable with therapeutic antibiotics, were isolated from the parotoid gland extract of the toad *Rhinella rubescens* [179]. The majority of molecules with antibacterial effects are AMPs derived from skin secretions, such as esculentins, brevinins, ranatuerins, ranacyclins, temporins, bombinins, and dybowskins. The skin secretion of *Ascaphus truei* contains eight peptides with broad-spectrum antibacterial effects called ascaphins [180]. Other molecules include syphaxin from *Leptodactylus syphax* [181], brevinvin from *Limnonectes fujianensis* [182], maximins from *Bombina maxima* [183], megins 1 and 2 from *Megophrys minor* [184], temporins from *Ranidae* family [185], etc. Their MIC values against both Gram-positive and Gram-negative species show promising results, suggesting their potential use in antimicrobial therapy, similar to brevinin-2 [182].

*Takifugu rubripes*, commonly known as the pufferfish, accumulates the potent neurotoxin tetrodotoxin (TTX) by consuming TTX-containing organisms within its food chain. This potent sodium channel blocker exhibits antibacterial effects against both Gram-positive bacteria like *E. faecalis* and Gram-negative bacteria, including *E. coli* [186].

## 6. Natural Food Products as Antimicrobial and Immune-Boosting Agents

Food-based antibacterial compounds are classified as antibacterial agents derived from plants or animals. Natural foods have long been valued for their therapeutic and nutritional benefits, offering a sustainable approach to health management. Extensive scientific investigations have revealed the bioactive components within these products, such as sulfur compounds, polyphenols, flavonoids, essential oils, alkaloids, and tannins, as well as AMPs, which contribute to antimicrobial, antiviral, anti-inflammatory, and antioxidant activities.

Garlic (*Allium sativum*), another well-researched antimicrobial food, contains allicin, a sulfur compound known for its antibacterial efficacy. Clinical studies indicate that allicin supplementation increases the eradication rate of *H. pylori*, facilitates ulcer healing, and alleviates associated symptoms [187]. In a clinical trial involving 15 patients, the Urease Breath Test (UBT) confirmed that garlic extract significantly reduced *H. pylori* colonization, supporting its potential as a complementary therapy [188]. Turmeric (*Curcuma longa*) also possesses strong antibacterial activity, primarily due to its bioactive compound curcumin. In antibacterial assays, turmeric fractions were tested against various bacterial strains, including *Bacillus cereus*, *Bacillus coagulans*, *Bacillus subtilis*, *S. aureus*, *E. coli*, and *P. aeruginosa*. A specific fraction eluted with 5% ethyl acetate in hexane exhibited the highest antimicrobial activity, reinforcing turmeric’s role as a potent antibacterial agent [189]. Cranberry (*Vaccinium macrocarpon*) has also been widely studied for its antimicrobial potential, particularly in the prevention of UTIs [190]. Proanthocyanidins in cranberry prevent bacterial adhesion to urinary tract epithelial cells, thereby reducing the recurrence of UTIs in otherwise healthy women. A systematic review and meta-analysis of seven randomized controlled trials suggested that cranberry supplementation is effective in preventing UTI recurrence; however, larger, high-quality studies are needed to confirm these findings [190].

Despite the promising antimicrobial properties of food-derived compounds, their widespread clinical application faces challenges related to standardization, regulatory approval, and quality control. Ensuring consistency in bioactive composition, establishing appropriate dosages, and addressing regulatory limitations are critical for integrating these natural antimicrobials into medicine. Scientific research plays a crucial role in regulatory frameworks, helping to navigate these challenges. Dwyer et al. provide a comprehensive overview of this issue in their review article, illustrating it with examples, such as a case study from the Office of Dietary Supplements at the National Institutes of Health (USA) [191]. This case study highlights important regulatory challenges and offers valuable resources for researchers. Additionally, the review discusses various regulatory obstacles and presents accessible tools for those seeking to deepen their understanding of the topic [191].

Natural products, such as honey and manuka honey, offer promising solutions. Honey is of plant origin and is produced by bees from flower nectar. Honey’s antimicrobial properties stem from its high sugar content, low pH, and the presence of hydrogen peroxide. Manuka honey, enriched with methylglyoxal (MGO), is particularly effective against antibiotic-resistant bacteria, including MRSA [192,193], and in promoting wound healing. Its bioactive compounds, including phenolics and flavonoids, disrupt bacterial membranes and inhibit metabolic functions. Manuka honey also demonstrates antifungal activity and efficacy against biofilm-forming bacteria like *P. aeruginosa* [194]. The low pH of honey is directly responsible for preventing bacterial growth in undiluted honey [195]. However, the pH increases when honey is ingested. On the other hand, diluting honey activates the enzyme glucose oxidase, which is naturally present in an inactive form at low pH. When honey is diluted, glucose oxidase is activated and acts on glucose to produce H_2_O_2_, with the highest level of hydrogen peroxide production occurring when honey is diluted by 30–50%, where the maximum antibacterial effect is observed [195]. Among the proteins in honey, defensin-1 is the primary component responsible for its antibacterial activity [195].

## 7. Antimicrobial Peptides (AMPs) and Peptidomimetics

**Figure 3:**
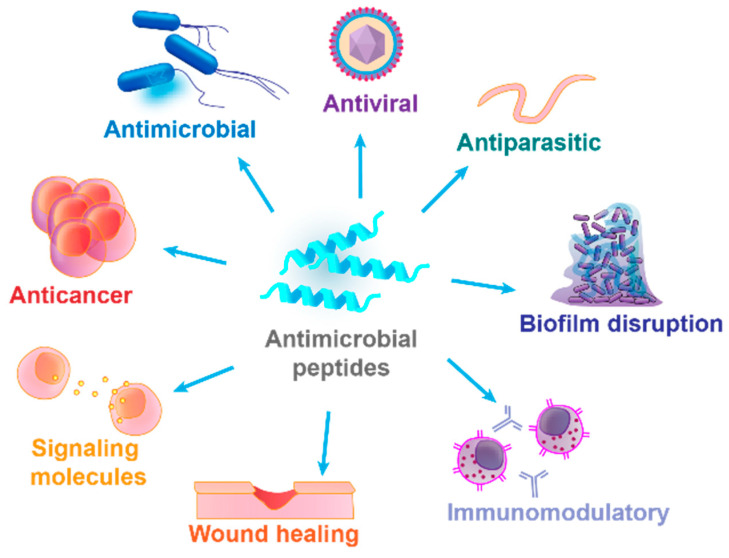
Various potential applications of antimicrobial peptides.

Short, often cationic and amphipathic molecules produced by various organisms, including bacteria, plants, animals, and humans, as part of their innate immune system, are referred to as AMPs. They possess potential for a wide range of applications in (i) medicine: the ability to destroy or inhibit the growth of various microorganisms, including bacteria, viruses, fungi, and parasites; wound healing; biofilm disruption; cancer therapy, etc.; (ii) agriculture; (iii) the food industry; (iv) the cosmetic industry; and (v) biomaterials (Figure 3).

AMPs typically consist of 12–50 amino acids, are mainly positively charged, and are amphipathic, meaning they have both hydrophilic and hydrophobic regions that enable interaction with microbial membranes. They act by directly interacting with the membranes of microorganisms, leading to pore formation and membrane permeabilization. They can also disrupt DNA replication, protein synthesis, and cellular metabolism. A key characteristic of AMPs is their low likelihood of resistance because their mechanisms of action make it difficult for microorganisms to develop resistance [196,197].

**Figure 4:**
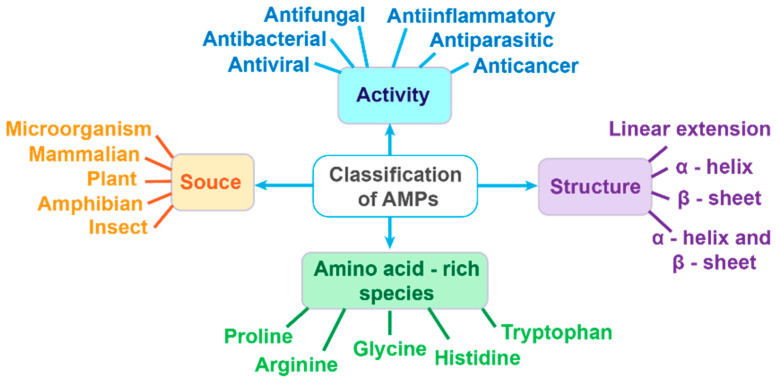
Classification of antimicrobial peptides (AMPs) based on source, activity, structure, and amino acid-rich species.

The diversity of natural AMPs makes their classification quite challenging; however, they can be classified based on (1) origin, (2) activity, (3) structural characteristics, and (4) amino acid-rich species (Figure 4) [198].

AMPs have great potential in the fight against pathogens; however, their use also comes with limitations. The key prerequisite for the clinical application of any drug, including AMPs, is that the benefits for a given indication outweigh the risks of possible adverse effects (sometimes including a manageable level of toxicity). In this regard, toxicity represents a major challenge for the clinical use of AMPs. It results from their ability to damage not only bacterial cell membranes but also those of host cells. For example, polymyxins (colistin, polymyxin B), which are currently used against multidrug-resistant bacteria, can cause nephrotoxicity and neurotoxicity [199]. Furthermore, the cationic and hydrophobic components of these peptides can directly interact with host cell components (e.g., red blood cells, mitochondria). Finally, some AMPs can trigger an excessive immune response, which may reduce their effectiveness or cause adverse reactions. Additionally, while the risk of developing resistance to AMPs is lower than that associated with antibiotics, evidence suggests that bacteria can develop adaptive mechanisms, such as changes in membrane structure or the active efflux of AMPs from the cell [200]. Finally, it should be noted that the synthesis and purification of AMPs are costly processes, making their commercialization more challenging compared to conventional antibiotics. However, research is being conducted to develop new methods that will reduce these costs [201,202].

Apart from toxicity, the stability of AMPs also represents a key challenge in their clinical application, which is why only a relatively small number of AMPs have successfully reached clinical use. One of the main limitations is the discrepancy between their in vivo and in vitro activity. Factors contributing to the low bioavailability of AMPs in vivo include the presence of numerous proteases and peptidases that can degrade AMPs. Consequently, their rapid degradation necessitates frequent administration. Additionally, the loss of activity under physiological conditions means that some AMPs lose efficacy in the presence of salts, serum, or low pH. For example, peptides such as pexiganan, iseganan, neuprex, and omiganan, which had shown great promise, failed in Phase III clinical trials due to low in vivo efficacy [201,203].

Peptidomimetics are molecules that mimic the structure and function of peptides but are usually more stable, less susceptible to enzymatic degradation, and often exhibit better bioavailability than natural peptides. They are not synthesized naturally in the body but are created using protein engineering technologies. Peptidomimetics are designed to replicate the biological activity of peptides while overcoming certain limitations associated with peptide use in therapeutic applications, such as poor oral absorption and rapid degradation by proteases. They can be used for drug design, cancer therapy, and as mimics of AMPs, making them potential treatments for bacterial, viral, or fungal infections. Furthermore, since they can cross the blood-brain barrier and target pathways associated with neurodegeneration, they are also being explored in this field [204,205].

Compared to natural AMPs, peptidomimetics offer several advantages that make them more suitable for clinical application. They can be chemically modified to resist enzymatic degradation, which increases their stability under physiological conditions. Additionally, they can be designed to selectively target bacterial cell membranes while reducing interactions with host cells, thereby minimizing unwanted effects such as nephrotoxicity and neurotoxicity [206].

Peptidomimetics can be optimized for improved absorption, distribution, and retention in the body, making them more effective for treating systemic infections. Their synthetic nature allows for continuous modifications, making it more difficult for bacteria to develop resistance. Additionally, peptidomimetics can be synthesized using more cost-effective chemical methods, allowing for easier large-scale production and lower manufacturing costs compared to AMPs [207].

Peptidomimetics represent a promising alternative to natural AMPs, addressing key issues such as stability, toxicity, and high production costs. However, challenges related to design and clinical validation still remain. With continued research, peptidomimetics have the potential to become a new class of effective antimicrobial agents, particularly in the fight against multidrug-resistant bacteria. Various methodologies and strategies have been developed and continue to evolve to establish systematic tools for transforming peptides into peptidomimetics or further into small drug-like molecules [205,208].

## 8. Natural Mineral Compounds

**Figure 5:**
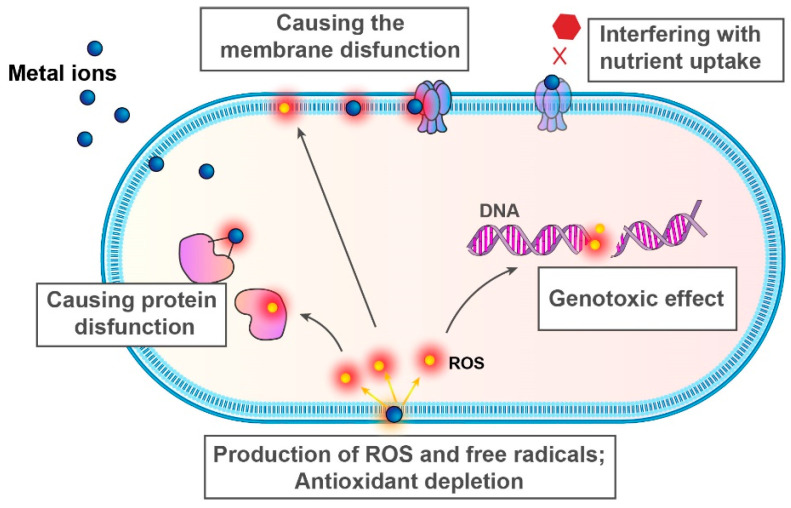
Mechanisms of antimicrobial effects of metals and metalloids; based on [209].

In low concentrations, certain metals and metalloids are essential for the functioning of all living cells but exhibit significant toxic effects when present in higher amounts [209,210] (Table 3; Figure 5).

Furthermore, some non-essential metals, such as silver, gold, and mercury, show toxicity to microorganisms at very low, nanomolar concentrations [210].

The antimicrobial effect of metals and metal-containing compounds has been known since ancient times, and before the era of antibiotics, some metals (most notably silver and copper) were common constituents of antimicrobial materials [210,211,212]. The modern era has brought a greater understanding of the possible mechanisms of action of metals in bacterial cells, which include the induction of membrane and protein dysfunction, the production of reactive oxygen species (ROS) and free radicals, and genotoxic effects (Figure 5).

Different metal alloys, salts, ions, various inorganic and organic complexes, as well as metallic nanoparticles, are currently being used as single antimicrobial agents to improve bioavailability and biocompatibility, or as adjuvants to antibiotics, for coating/integral components of biomedical devices and biomaterials, or even in the imaging of infected tissues [209,210,212].

Maybe one of the most explored systems are those based on silver [213]—silver enhances the activity of antibiotics and even sensitizes some antibiotic-resistant bacteria [211]. Silver nanoparticles have become widely used as antimicrobial agents, along with other types of metallic nanomaterials [214]. Further promising agents are the organic metal complexes—metallophores, which improve the delivery of antimicrobials and enable bacteria to acquire essential metals through similar organic complexes (such as siderophores) [209,210,212]. Additionally, light-activated (photoactivated) metallic complexes [210,212], as well as synthetic mineral compounds, such as clays, have shown promise [215].

## 9. Nanoformulations

The application and biological activities of many antimicrobial agents have been greatly improved through the use of nanotechnology. This technology provides control over a range of physicochemical properties of materials, which are directly responsible for biological effects. Nanomaterials can be used in the prevention, treatment, and diagnostics of infectious diseases. While some antimicrobial nanomaterials possess direct antimicrobial activity, others serve as systems for improved delivery of antimicrobials, such as antibiotics.

### 9.1. Nanoparticles as Antimicrobial Agents

Regarding direct antimicrobial activity, nanoparticles with smaller sizes and positive surface charges usually achieve more pronounced effects. Also, they can pass through biological barriers such as biofilms. For this purpose, metal and metal oxide nanoparticles are most often used, especially those made of silver, gold, copper, ZnO, TiO_2_, MgO, etc. [214].

### 9.2. Nano-Carriers of Antimicrobial Agents

On the other hand, many nanomaterials serve as transport systems for active substances. Among them, polymeric nanoparticles and liposomes are commonly used in the drug delivery of antibiotics, lowering the required concentration and reducing toxicity to human cells [216]. In addition to antibiotics, other agents with antimicrobial activity, such as natural compounds, can also be applied more efficiently as part of nanosystems.

Finally, the versatility of methods in nanotechnology allows for numerous combinations of different agents, often with the aim of achieving synergistic effects.

### 9.3. Issues Regarding the Use of Antimicrobial Nanomaterials

With many novel agents intended for widespread use, several issues are coming to the forefront. A priority is ensuring product safety, especially regarding metallic nanoparticles [217]. A detailed assessment, particularly regarding the mechanism of action and metabolism in humans, is necessary before the clinical application of any of these experimental agents, although some of them, like zinc, silver, and gold nanoparticles, have already successfully passed the FDA approval process [218]. Additionally, the EMA has issued four guidelines on nanomedicines covering the development and evaluation of nanoparticle-based drugs (https://www.ema.europa.eu/en/human-regulatory-overview/research-and-development/scientific-guidelines/multidisciplinary-guidelines/multidisciplinary-nanomedicines, accessed on 16 February 2025). It has also approved solid lipid nanoparticles, such as liposomal amikacin, for treating lung infections caused by *Mycobacterium avium* in adults with limited treatment options and without cystic fibrosis (https://www.ema.europa.eu/en/medicines/human/EPAR/arikayce-liposomal, accessed on 16 February 2025). Another issue is the possibility of bacterial resistance. Although it is less likely to happen in case of agents that have several mechanisms of action, such as metallic nanoparticles, it can still occur if significant selective pressure is present. Bacterial resistance to silver, caused by the large amounts of silver and silver nanoparticles released into the environment, has already been documented [219].

## 10. Conclusions and Future Directions

The increasing prevalence of AMR represents a pressing global health challenge, emphasizing the urgent need for novel therapeutic strategies. In response, natural antibacterial agents sourced from microorganisms, plants, animals, natural minerals, and even food-based substances have emerged as promising alternatives to conventional antibiotics. These natural compounds exhibit a diverse range of mechanisms that show significant potential in combating MDR pathogens, positioning them as important players in the battle against AMR.

Therefore, this review highlights the antibacterial properties of well-known natural agents (such as antibiotics), promising complementary substances (bacteriocins, plant-based compounds), and even unconventional sources like snake venoms and microbiopredators. These new agents differ in their mechanisms of action from traditional antibiotics, with effects ranging from direct bactericidal actions to the modulation of host immune responses. This diversity contributes to their potential significance in treating infections caused by antibiotic-resistant bacteria.

Certain natural antibacterial options, such as phages, probiotics, and some plant-based products, have already undergone clinical trials, while others, like mushroom-derived agents, remain in the early stages of investigation. Despite their promising features, rigorous testing methods and clinical validation are critical to ensure their safety and efficacy. Continued research focused on improving extraction and/or concentration methods, standardizing dosage/effects, and developing modification techniques may ultimately make it possible for these natural agents to be integrated into future anti-infective treatments and clinical practice.

While fundamentally different from one another and from antibiotics (e.g., the higher specificity of phages, concerns over the stability and toxicity of AMPs, and the inconsistent clinical validation of plant-based compounds), they show significant potential to complement and improve current treatment protocols. However, it remains uncertain whether they will eventually be able to replace antibiotics in the future.

Further research should prioritize the development of environmentally friendly and sustainable methods for sourcing natural antibacterial agents, ensuring that their extraction does not negatively impact ecosystems or lead to resource depletion. At the same time, improving drug formulations, particularly through innovative delivery systems like nanoparticles, could enhance the bioavailability and effectiveness of these compounds, making them more efficient in the body. Additionally, combining natural antibacterial agents with traditional antibiotics may help mitigate the risk of resistance, thereby improving treatment outcomes. Another promising area is the exploration of targeted therapies, which could focus on specific bacterial strains or resistance mechanisms, thereby maximizing the therapeutic potential of natural agents in personalized medicine.

To fully integrate these agents into clinical practice, extensive clinical trials are necessary to validate their safety and efficacy. The development of clear regulatory frameworks will be essential for defining the standards and the appropriate pathway for their approval and broader use in healthcare settings. Furthermore, mining novel species from previously uncharted territories on land and sea remains vital for discovering new classes of antibiotics.

Through these efforts, natural antibacterial agents may have the potential to complement existing antibiotic-based treatments, playing an indispensable role in combating AMR in a sustainable and effective manner.

## Figures and Tables

**Table 1:** Frequently used antibiotics, categorized by their microbial origin, along with their year of discovery, initial medical application, and indications.

Antibiotic	Origin	Year of Discovery	Introduced for Use	Indications
Penicillin	*Penicillium notatum* (now *Penicillium chrysogenum*)	1928	1940	Gram-positive bacteria (pharyngitis, pneumonia), syphilis.
Cephalosporins	*Acremonium* spp.	1945	1964	Five distinct generations, each with a varying range of activity.
Thienamycin (carbapenem)	*Streptomyces cattleya*	1976	1970s	MDR Gram-negative bacteria
Streptomycin	*Streptomyces griseus*	1943	1944	The first effective treatment for TB. Also effective in treating other bacterial infections like plague and brucellosis.
Actinomycin	*Streptomyces species* (primarily *Streptomyces parvullus*)	1940s	1950s	The first compound, actinomycin D, is primarily used in cancer treatment due to its ability to stop rapidly dividing cells.
Tetracyclines	*Streptomyces aureofaciens* and other *Streptomyces* species	1948	1950s	Broad-spectrum antibiotics. Tetracyclines have been used to treat conditions like acne, urinary tract infections, and respiratory infections.
Chloramphenicol	*Streptomyces venezuelae*	1947	1949	Broad-spectrum antibiotic
Vancomycin	*Amycolatopsis orientalis* (formerly *Streptomyces orientalis*)	1953	1958	Active against Gram-positive bacteria, including *Staphylococcus aureus* (especially MRSA). Due to its effectiveness against MDR strains, it is often referred to as a “last-resort” antibiotic.
Rifamycin	*Amycolatopsis rifamycinica* (formerly *Streptomyces mediterranei*)	1957	1970s	Primarily used to treat tuberculosis and leprosy, as well as other bacterial infections, such as those caused by *Staphylococcus* spp.
Erythromycin	*Saccharopolyspora erythraea* (formerly *Streptomyces erythraeus*)	1952	1950s	Active against Gram-positive bacteria and some Gram-negative bacteria. It is particularly useful for treating respiratory infections, skin infections, and sexually transmitted diseases. Erythromycin is considered an alternative for individuals allergic to penicillin.
Polymyxins	*Bacillus polymyxa* (and related species)	1947	1950s	They are particularly effective against MDR bacteria like *Pseudomonas aeruginosa*, *Escherichia coli*, and *Klebsiella* spp. While polymyxins (especially polymyxin B and colistin) were once widely used, their toxicity (nephrotoxicity and neurotoxicity) has limited their usage. However, they have experienced a resurgence in clinical application.

MRSA: methicillin-resistant *Staphylococcus aureus*; TB: tuberculosis; MDR: multidrug-resistant. According to [10].

**Table 3:** Essential metals and metalloids with significant antimicrobial activity (based on [210]).

Metals Essential for All Organisms	Antimicrobial Activity of Metal(oid)s—Range of Efficient Concentrations		
	Nanomolar	Micromolar	Millimolar
Manganese	Tellurium TeO_3_^2−^	Copper Cu^2+^	Cobalt Co^2+^
Iron	Mercury Hg^2+^	Zinc Zn^2+^	Aluminum Al^3+^
Cobalt	Silver Ag^+^	Nickel Ni^2+^	Gallium Ga^3+^
Nickel	Gold Au^3+^	Bismuth Bi^3+^	Tungsten WO_4_^2−^
Copper			Manganese Mn^2+^
Zinc			Selenium SeO_3_^2−^

## Data Availability

Not applicable.

## Abbreviations

The following abbreviations are used in this manuscript:

AMR	Antimicrobial resistance
AMP	Antimicrobial peptide
AST	Antimicrobial susceptibility testing
BV	Bacterial vaginosis
CDAD	*Clostridioides difficile*-associated diarrhea
CFU	Colony forming unit
CRAB	Carbapenem-resistant *Acinetobacter baumannii*
CRPA	Carbapenem-resistant *Pseudomonas aeruginosa*
DNA	Deoxyribonucleic acid
EHEC	Enterohemorrhagic *Escherichia coli*
EMA	The European Medicines Agency
EO	Essential Oil
ESKAPEE	*Enterococcus faecium*, *Staphylococcus aureus*, *Klebsiella pneumoniae*, *Acinetobacter baumannii*, *Pseudomonas aeruginosa*, *Enterobacter* spp., and *Escherichia coli*
EU	European Union
FDA	Food and Drug Administration
GRAS	Generally recognized as safe
L-AAO	L-amino acid oxidase
LLE	Liquid–liquid extraction
MAE	Microwave-assisted extraction
MBL	Metallo-β-lactamases
MDR	Multidrug-resistant
MGO	Methylglyoxal
MIC	Minimum inhibitory concentration
MRSA	Methicillin-resistant *Staphylococcus aureus*
NIH	National Institutes of Health
PEF	Pulsed electric field extraction
PLA2	Phospholipase A2
QS	Quorum system
ROS	Reactive oxygen species
SFE	Supercritical fluid extraction
TB	Tuberculosis
TTSS	Type three secretion system
TTX	Tetrodotoxin
UAE	Ultrasound-assisted extraction
UBT	Urease Breath Test
US	United States
UTI	Urinary tract infections
VRE	Vancomycin-resistant *Enterococcus faecalis*
VRSA	Vancomycin-resistant *Staphylococcus aureus*
WHO	World Health Organization
